# A Critical Review: Recent Advancements in the Use of CRISPR/Cas9 Technology to Enhance Crops and Alleviate Global Food Crises

**DOI:** 10.3390/cimb43030135

**Published:** 2021-11-11

**Authors:** Adnan Rasheed, Rafaqat Ali Gill, Muhammad Umair Hassan, Athar Mahmood, Sameer Qari, Qamar U. Zaman, Muhammad Ilyas, Muhammad Aamer, Maria Batool, Huijie Li, Ziming Wu

**Affiliations:** 1 Key Laboratory of Crops Physiology, Ecology and Genetic Breeding, Ministry of Education/College of Agronomy, Jiangxi Agricultural University, Nanchang 330045, China; adnanbreeder@yahoo.com (A.R.); lihuijie169@163.com (H.L.); 2 Oil Crops Research Institute, Chinese Academy of Agricultural Sciences, Wuhan 430062, China; drragill@caas.cn (R.A.G.); qamaruzamanch@gmail.com (Q.U.Z.); 3 Research Center on Ecological Sciences, Jiangxi Agricultural University, Nanchang 330045, China; muhassanuaf@gmail.com (M.U.H.); muhammadaamer@jxau.edu.cn (M.A.); 4 Department of Agronomy, University of Agriculture, Faisalabad 38040, Pakistan; athar.mahmood@uaf.edu.pk; 5 Biology Department, (Genetics and Molecular Biology Central Laboratory), Aljumum University College, Umm Al-Qura University, Makkah 24382, Saudi Arabia; shqari@uqu.edu.sa; 6 University College of Dera Murad Jamali, Nasirabad 80700, Balochistan, Pakistan; muhammadilyas.ucdmj@luawms.edu.pk; 7 College of Plant Science and Technology, Huazhong Agricultural University, Wuhan 430070, China; maria.batool@webmail.hzau.edu.cn; 8 College of Humanity and Public Administration, Jiangxi Agricultural University, Nanchang 330045, China

**Keywords:** CRISPR/Cas9, crops, yield, biotic, abiotic stresses

## Abstract

Genome editing (GE) has revolutionized the biological sciences by creating a novel approach for manipulating the genomes of living organisms. Many tools have been developed in recent years to enable the editing of complex genomes. Therefore, a reliable and rapid approach for increasing yield and tolerance to various environmental stresses is necessary to sustain agricultural crop production for global food security. This critical review elaborates the GE tools used for crop improvement. These tools include mega-nucleases (MNs), such as zinc-finger nucleases (ZFNs), and transcriptional activator-like effector nucleases (TALENs), and clustered regularly interspaced short palindromic repeats (CRISPR). Specifically, this review addresses the latest advancements in the role of CRISPR/Cas9 for genome manipulation for major crop improvement, including yield and quality development of biotic stress- and abiotic stress-tolerant crops. Implementation of this technique will lead to the production of non-transgene crops with preferred characteristics that can result in enhanced yield capacity under various environmental stresses. The CRISPR/Cas9 technique can be combined with current and potential breeding methods (e.g., speed breeding and omics-assisted breeding) to enhance agricultural productivity to ensure food security. We have also discussed the challenges and limitations of CRISPR/Cas9. This information will be useful to plant breeders and researchers in the thorough investigation of the use of CRISPR/Cas9 to boost crops by targeting the gene of interest.

## 1. Introduction

The global population continues to grow at an alarming rate [1], but there is an arithmetic increase in the number of food materials available [2]. The global population is expected to increase to 10 billion by 2050 [3,4]. The global population and the negative impact of climatic conditions may eventually create issues regarding food security. The condition may be further aggravated by a decline in the productive areas and reduced yield. Recent estimates from the International Rice Research Institute (IRRI) have indicated that every 7.7 s, one hectare of the fertile area is lost, and the effect may be more pronounced if the global temperature acceleration rate persists [5]. To maintain food security, crop yield capacity must nearly double, and highly tolerant cultivars against various stresses must be developed to achieve this goal [6].

An increase in agricultural crop production using the latest breeding techniques [7] is the primary concern regarding global food security [8]. However, conventional breeding techniques cannot ensure food safety and maximum food production [9,10,11,12]. Varietal improvement and crop development have been accomplished using traditional breeding techniques, such as hybridization, which to some extent, has enhanced the production of crops [13,14,15]. Genetic engineering has been the focus of research for several years in determining the function of genes. This involves using physical and biological mutagenesis and identification of biological contrivances to boost crop production. Owing to several issues, a 100% success ratio cannot be achieved [16]. In recent years, the actual yield has been recorded for the major cereal crops, such as rice in East Asia and wheat in Northwest Europe [17]. Additionally, aspects of the introduction of transgenes into the plant host genome raise public concerns regarding edible crops and are not always appreciated, owing to a lack of clarity on the methods used and consequent benefits. Therefore, the use of biotechnological tools for crop enhancement is of ultimate significance in overcoming these limitations [17].

**Figure 1:**
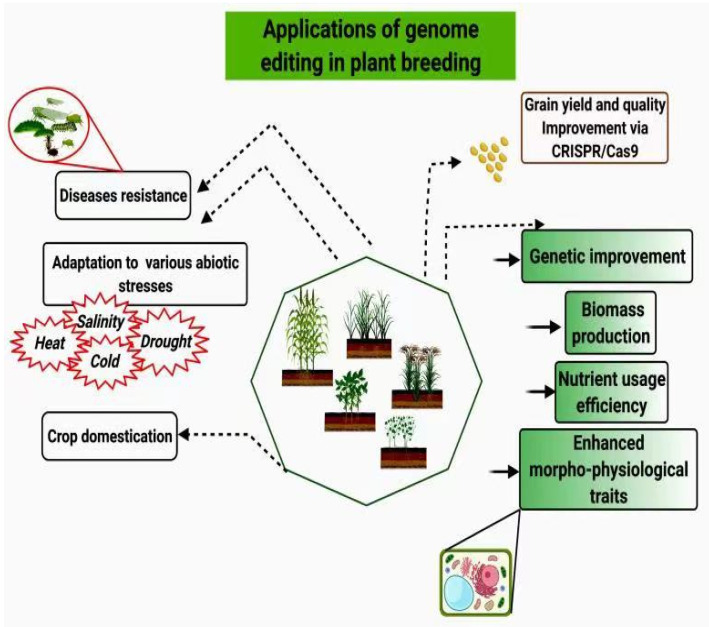
Application of genome editing in crops improvement. CRISPR/Cas9 is used for genetic improvement, increasing nutrients use efficiency, biomass production, and increasing disease resistance.

Genetic engineering includes several methods, such as zinc-finger nucleases (ZFNs), transcriptional activator-like effector nucleases (TALENs), and CRISPR/Cas9 (clustered regularly interspaced short palindromic repeats) [18]. ZFNs are defined as DNA cleavage proteins designed to split DNA structures at a given location. Second, TALENs generate breaks in double strands of targeted structures, activating the DNA response pathway that leads to genome breakage [19]. Although ZFNs and TALENs are commonly used for genome alterations in plants and animal cells (since 2002 and 2011, respectively), a few limitations exist that impede their successful use. The specificity of ZFNs is small and often results in off-target changes [20]. Construction of vectors is time and laborconsuming for ZFNs and TALENs [21]. Therefore, continuous progress in crop breeding programs would be vital in addressing these tasks and for developing food crops [22]. Recent developments in CRISPR/Cas9 tools have rendered directed and accurate genetic alteration of crops possible, increasing the speed of transition to crop improvement [23]. Only a few species have been studied thus far using this approach [24]. Since 2013, the focus shifted to the use of CRISPR/Cas9 and its variants. CRISPR is a DNA fragment that includes non-contiguous, small DNA repeats interspaced by variable sequence fragments or spacers. Therefore, this finding indicated that CRISPR/Cas9 could be responsible for the elicitation of adaptive immunity in prokaryotes [25,26] ISPR/Cas9 has the potential to cure diseases [27], and the use of this technique has a huge impact on plant biology [28]. The first reported application of CRISPR/Cas9 is an adaptive immune mechanism described in an experiment in 2007, wherein investigators considered the phenotype to be phage-resistant, and the addition or removal of specific spacers could alter the bacterial phenotype [29]. In the future, it will be easy to genetically alter plants for crops improvement [30]. The use of Cas9 (a protein known as biological scissors) sets new standards and revolutionizes genome editing, which will lead to new insights into agricultural crops improvement [31]. Here, we briefly reviewed the role of CRISPR/Cas9 applications in crop improvement, its limitations, and future prospects. CRISPR/Cas9 is currently being used for different aspects in plant breeding like, diseases resistance, drought resistance, salinity tolerance and genetic improvement of crops for various aspects. The grain yield and quality of crops is also improved by Cas9. The production of biomass has also been improved by using Cas9. Likewise, nutrients use efficiency and enhancement of morpho-physiological traits has been done using Cas9. Role of genome editing is shown in Figure 1.

## 2. Genome Editing Tools

Genome editing aims to revolutionize plant breeding and could help safeguard the global food supply chain [32]. Meganucleases are first-generation gene-editing tools and [33] were the first tools used for genome editing and have been classified by the presence of an approximately 12–40 bp recognition site. They are often known as the most defined restriction enzymes because of their precise nature and broad recognition site [34]; hence, they are also called homing endonuclease enzymes. Research studies have shown that the repair process of double-stranded breaks is caused by non-homologous end joining (NHEJ), responsible for the knockout of genes present in *Arabidopsis* and tobacco [35,36]. As DNA binding domains are attached to a catalytic area of meganucleases, separation is not possible, and therefore it is practically impossible to alter the meganucleases with other genome editing techniques [37]. There are several examples of studies in which meganucleases (MNs)were exploited in cotton [38] and maize [39] using alerted MNs; however, studies are warranted to enhance the methodology.

The second gene-editing tool includes ZFNs, which are more efficient and reliable owing to the generation of double-stranded breaks (DSBs) [40]. ZFNs were used as the first genome manipulation approach developed by the application of engineered nucleases. Detection of the Cys2-His2 Zinc-finger domain made this approach possible [19]. The structure of ZFNs consists of the following two main domains: (1) the DNA-binding domain containing 300 to 600 zinc-finger repeats [41]. Individually zinc-finger repeats can be observed between 9–18 bp; and (2) the DNA slice domain, considered a nonspecific domain of type 2 restriction endonucleases [42]. ZFNs comprise two monomers accredited to their particular object sequences reversely adjoining 5- and 6-bp DNA targets [43]. DNA is sliced by dimer enzymes comprising the FokI domain. The specific order of 24 to 30 bp is read by a zinc-finger domain that contains particular or occasional directing positions in the genome [44]. The genome-editing field has shown success and advancements in acquiring the capacity to control and alter the basic genomic marks.

TALENs have been widely used for many years. TALENs were developed by consolidating the FokI slice domain to the DNA-binding domains of the TALE proteins. These TALEs contain complex duplications of 34 amino acids for the effective alteration of a single base pair [45]. TALENs also cause modifications by creating DSBs that trigger initiation of pathways where editing occurs [19]. The central domain and the nuclear localization sequence are present in the TALEN system [46]. The proficiency of proteins to alter DNA was studied for the first time in 2007. Nevertheless, the DNA-binding domain comprises 34 amino acid repeat sequences, and a single nucleotide is detected in the DNA target region by each repeat, whereas individual constant sequences of ZFNs are observed as three nucleotides in the targeted DNA [47]. Few studies have been conducted using TALENs and ZFNs in plants; although these studies favor the application of TALENs, their gene-editing effectiveness is usually low. TALENs are more effective and reliable for genome editing programs [44]. A list of different crop traits improved by the usage of MNs, ZFNs, and TALENs is shown in Table 1.

### 2.1. CRISPR/Cas9 and Its Brief Overview

CRISPR/Cas9 genome editing technique is definedas clustered regularly interspaced short palindromic repeats, short, repeating variants of genetic material and found in most archaea as well as in many bacterial species. CRISPR/Cas9 and its related proteins develop a very strong protective system that safeguards plants against foreign agents like viruses, bacteria, and other elements. CRISPR/Cas9 is often used to mutate the targeted genes within the system [56] ISPR/Cas9 was discovered in DNA cleavage systems programmed by RNA, which has been found in bacteria and archaea. CRISPR/Cas9 is the most commonly used and well-studied CRISPR/Cas9 system [57,58]. Current recognition of the researchers for the development of a genome editing method using CRISPR/Cas9 by the Nobel Prize committee is an additional step nearer to developing and cultivating novel varieties of crops [59]. The system comprises two constituents, namely an endonuclease DNA (Cas9) and a target-specific RNA, called the single guide RNA (sgRNA) [57,58]. The basic steps to use Cas9 involves the existence of a protospacer adjacent motif (PAM) sequence near and directly pointed to a given target. Various spacer sequences are necessary for the application of Cas9 as a target; therefore, CRISPR/Cas9 is fast, effective, cost-effective, and adaptable. Parts of CRISPR/Cas9, either in DNA or RNA, are administered to plant cells for the CRISPR/Cas9-mediated manipulation of genomes to cut plant DNA in a predetermined series. In this manner, plant cells initiate processes to “patch” the break to maintain genome integrity, and this hints at the generation of numerous forms of alterations in the directed sequence. When the break is fixed via NHEJ/homology-directed repair (HDR), small deletions or insertions occur that can lead to the mutation of genes.

Otherwise, the accessibility of a homologous DNA template present around the targeted point can cause an HDR, which results in the insertion of a DNA template, thereby allowing accurate gene replacement or insertion [60]. Base manipulation is the newest addition to the features of this tool [61,62]. Until now, changes in A–G or C–T have been attained through the application of base manipulators [61,62], which has sparked significant interest in food crop improvement-related gene editors. The CRISPR/Cas9 technology can also be used with the dead Cas9/Cas12 a for gene control, epigenetic alteration, and chromosome imaging [23]. A comparison of the functions of different GE tools is described in Table 2. 

**Figure 2:**
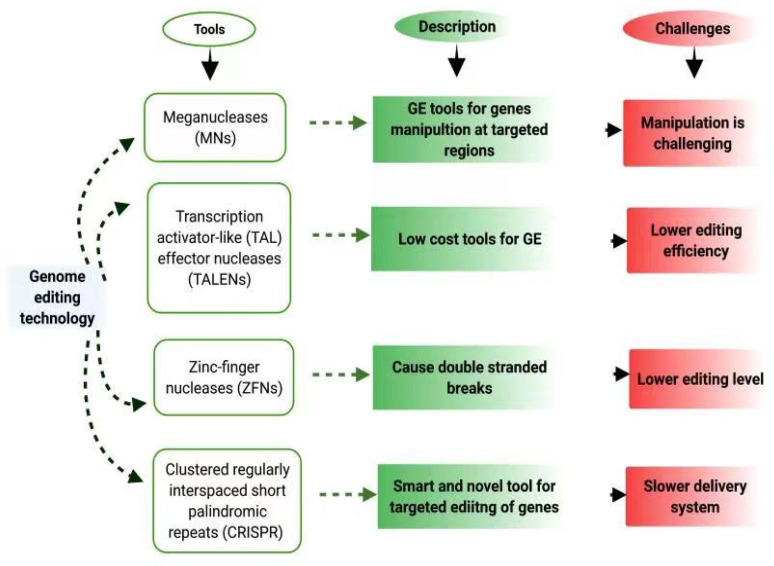
A comparison of genome editing tools. Comparison of these tools shows their efficiency towards genome editing and also shows their limitations. Hence CRISPR/Cas9 technique is more accurate than other tools.

Throughout the characterization of spacer absorption, motifs related to spacer precursors (protospacers), an invading bacteriophage, were isolated from DNA [25,63]. Such motifs are small extensions of trinucleotides, situated directly after the protospacers, which have a significant function in recognizing particular protospacers and in guiding the location of the spacers inserted in the protospacer repeat arrays [63]. Brouns, et al. [64] showed that a CRISPR RNA precursor transcribed from a CRISPR site was split into mature RNA molecules inside the repeated chain of proteins of Cas (to crRNA). Every crRNA comprises a spacer flanking short duplications of DNA and acts as a small RNA guide that helps proteins elicit an antiviral action [64]. CRISPR/Cas9 in *Streptococcus* thermophiles have been shown to slice plasmid DNA [65]. Such results support the molecular origin of adaptive immunity facilitated by the CRISPR/Cas9 system. Each of the genome-editing techniqueshas its advantages and drawbacks, as shown in Figure 2.

**Table 2:** Differences in functions of genome editing tools.

Role	ZFNs	TALENs	MNs	CRISPR/Cas9	References
Efficacy of target recognition	Higher	Higher	Higher	Higher	[66]
Kind of Action	Double-stranded break in target DNA	Double-strandedbreak in target DNA	Direct conversions in targeted regions	Double-stranded break in target DNA	[67]
Mutagenesis	Higher	Middle	Middle	Lower	[66]
Multiplexing	Difficult	Difficult	Difficult	Possible	[68,69]
Target range	Unlimited	Unlimited	Unlimited	Limited by PAM	[70]
Effects	Lower	Lower	Lower	Lower	[71]
Cost	Higher	Higher	Higher	Low	[72]
Crop Improvement	Low	Low	Low	Higher	[72]
Range	Narrow	Narrow	Narrow	Broad	[72]
Dimerization	Required	Not required	Not required	Not required	[71]
Types	One	One	One	Many	[71]
Future use	Medium	Medium	Medium	High	[72]

### 2.2. CRISPR/Cas9 Mechanism and Function

**Figure 3:**
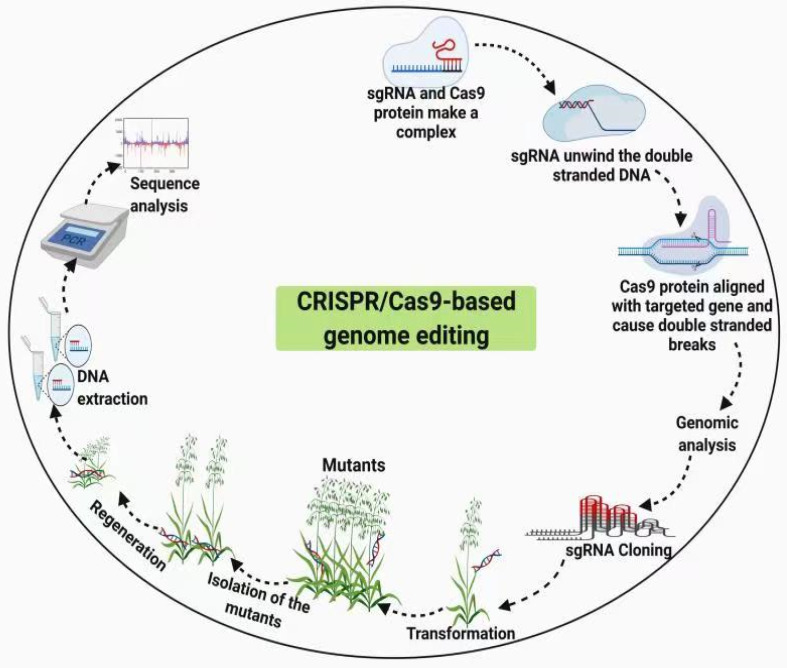
Mechanism of genome editing using Cas9. Cas9 gene-editing system involves complex of sgRNA and Cas9 protein, unwinding of DNA by sgRNA, cutting of gene by Cas9, use of analysis tools, cloning, and transformation, etc. This process needs no foreign element for editing.

Makarova, et al. [73] suggested that the immunity process comprises three phases facilitated by the CRISPR/Cas9 system, including adaptation, expression, and interference. In the adaptation phase, short plasmids are inserted as new spacers into the CRISPR/Cas9 series, whereas in the expression phase, crRNA transcription and maturation processes occur. Ultimately, the crRNAs direct Cas proteins in the interference process to match the splitters. Makarova et al. [73] suggested the hierarchical grouping of CRISPR/Cas into three main groups, namely I, II, and III, as described in a study on the origin of Cas proteins and CRISPR/Cas9. Type I and III systems involve the application of a complex of multiple Cas proteins as sign proteins, whereas Cas9 is a large multifunctional single sign protein in the Type II system and is accountable for both the development of crRNA and slicing of the target DNA [73]. The Type II system has a relatively easy architecture compared to that of the other systems and can be developed more effectively to act as a genome editing method. Mechanisms of genome editing using CRISPR/Cas9 involves, sgRNA and Cas9 make a complex, sgRNA unwinds DNA, and Cas9 cut the DNA, genome analysis, cloning of sgRNA, transformation and selection of plants, regeneration, extraction of genomic DNA and final step is analysis of sequence to confirm the results Figure 3.

Regarding the Type II system, a small trans-noncoding RNA (transactivating CRISPR/Cas9 RNA) was discovered in *S. pyogenes,* and it has been shown to direct the development of crRNAs through the pairing of bases with duplicated regions of transcripts of pre-crRNA via the action of RNase III and Cas9 [74]. Jinek et al. [57] found suggested application of a Type II system and synthesized a sgRNA via the fusion of crRNA and tracrRNA. The 5′ end 20-bp sgRNA sequence, a target binding DNA sequence, may be converted into any sequence [57]. The analysis described above indicated that three pairs of PAM bases [57,65] could be programmed with a sgRNA to produce DSB at a point from the nuclease of Cas9 from *Sogenes* (SpCas9). The sequencing and location of PAMs vary across various CRISPR/Cas systems [63]. A canonical PAM related to SpCas9 is 50-NGG-30 (N exemplifies one of the four nucleotides), and a 20bp target DNA sequence was accompanied by a PAM [57,63]. Cas9 nuclease comprises two domains identical to HNH and RuvC [73]. Jinek et al. [57] also found that the HNH and RuvC-like domains could complement the DNA strand and not the RNA reference, respectively. Therefore, this system is effective and programmable and acts similar to a model CRISPR/Cas9 system, which is most frequently used to alter genes, with the alterations facilitated by NHEJ/HDR. Single-guided RNA bioinformatics tools developed for CRISPR/Cas9 are listed in Table 3. The role of different RNAs in CRISPR/Cas9 is varied and highly studied. The guided RNA (mgRNA) is a particular RNA sequence thatidentifies the targeted regions of the DNA region and regulates the Cas9 nuclease enzyme for gene editing. Hence, it unwinds the DNA and aligns the gene with Cas9 protein, and causesdouble-stranded breaks. The second type of RNA is crRNA which describes the targeted DNA for Cas9protein, whereasthe role of tracrRNA is to work asa scaffold linking the crRNA molecule with Cas9 protein and assist insorting out of mature crRNAs from the pre-crRNAs developed from CRISPR/Cas9 arrays Table 3 [75]. 

## 3. Application of CRISPR/Cas9 for Crop Improvement

CRISPR/Cas9 technique has become the most widespreadand has many advantages, like time effectiveness, low cost of editing, extraordinary adaptability, and the ability for directing multiple genes reproductions instantaneously [86]. This gives extraordinary worth for breeding of numerous polyploid species of crops species, which are problematic to progress using classical approaches, and lets the group of a range of monoallelic as well as biallelic mutants, and a resulting allelic sequence of phenotypes, in the first generation, something that is characteristically not possible using classical breeding approaches. This technique is a more efficient and versatile technique of genome editing [87]. This technique, i.e., CRISPR/Cas9, is simple, efficient, and cost-effective relative to TALENS, ZFNs, and MNs, and it is used to edit multiple genomes at the same time [88,89]. Owing to its positive features, the CRISPR/Cas9 system has been used for several plant species [90,91], and its application provides the best solution to multiple issues encountered in plant breeding [92]. The CRISPR/Cas9 has been used to improve the monocots and dicots crops for a variety of traits like yield, quality, disease resistance, and resistance to climatic variations [93]. The CRISPR/Cas9 technique has been used to edit the genomes of cereal crops, such as wheat, maize, rice, cotton, and vegetables like tomatoes, and potatoes and fruits like banana and apple [94,95]. The most common use is the knockout of target genes that have been gained by induction of indels that result in a frameshift mutation. A large number of important traits have been introduced in crops using Cas9 and have been discussed in many reviews [96,97,98,99].

### 3.1. Use of CRISPR/Cas9 for Improvement of Yield and Quality

CRISPR/Cas9 creates revolutionary changes in crop species [100]. Several laboratories in the world have been established that use this powerful technique because of its potential. Here, we listed some of its uses that have been considered in the improvement of the yield and quality of crops. CRISPR/Cas9 is used to develop cultivars with high nutritional values and resistance characteristics and to create the largest milestone of genome editing in modern technology. A commercial oil was obtained from the crop seedsof *Camelina sativa*, which is widely cultivated because of its higher percentage of fatty acids. Moreover, the JAZI, a jasmonate-zim-domain protein, BRII brassinosteroid insensitive 1, and GA-insensitive genes were also altered, and CRISPR/Cas9 resulted in a 26% to 84% mutation frequency [17]. In the same species, a squamosal promotor binding protein and a flowering locus were manipulated using Cas9, and at the late flowering stage, the plants showed approximately a 90% mutation frequency [101]. A multiple CRISPR/Cas9 system combined with other tools was engineered and applied for the manipulation of six altered *PYL* genes of ABO receptors with a 13%–93% mutation frequency in the first generation [102]. The green fluorescent gene present in *Nicotiana benthamiana* with RNA-facilitated endonucleases was altered [90]. Later, this was delivered through the tobacco rattle virus to modulate the instructions to the plant genes to manufacture and manipulate transcriptional features [103].

The progenies of homozygous rice species were altered using CRISPR/Cas9, and the results showed a deletion in the gene cluster on the chromosome with heritable variations in genetic makeup [104]. Mutagenesis in three individuals in the aldehyde oxidase gene family of rice, *OsAox1b*, *OsAox1a*, and *OsAox1c*, in addition to the (abbreviation) *OsBEL* gene mediated by CRISPR/cas9, was observed. Subsequently, inherent alterations of genes in successive progenies were studied [105]. An ABA-inducible protein encoded by the barley gene *HvPM19* was responsible for the upregulation of genes responsible for grain dormancy. As a result of mutations induced by CRISPR/Cas9 in *HvPM19,* a 10% mutation frequency was generated [106]. The role of two QTLs, *Gn1a* and *GS3*, for rice grain number and size, were also investigated by Cas9 [107]. Usman, et al. [108] studied the editing of *OsSPL16* gene in rice by Cas9 and found an improvement in rice grain yield. *CLBG1* mutagenesis by Cas9 in watermelon significantly reduced seed size and enhanced seed germination [109]. Cas9 can be used to improve every trait in crops, and this could lead to a green revolution in time. The base editing of the *WAXY* allele of granule bound starch synthase improved the cooking quality of rice using Cas9 [110]. Zhang, et al. [111] studied the disruption of *MIR396f* and *MIR396e*,which improves the yield of rice under low nitrogen conditions. The grain yield modulator miR156 regulates the seed dormancy in rice as studied in an experiment [112]. In a recent study, new rice mutants with high yield and aroma were generated by editing of three homologs (*GL3.2*, *Os03g0568400*, and *Os03g0603100*) of *P450* and *OsBADH2* using CRISPR/Cas9 in rice [113]. A high content of protein often reduces the rice cooking quality. By targeted mutagenesis of transporter genes of amino acids could bring significant variation among the mutants. The *OsAAP6* and *OsAAP10* are two mutants generated in rice high-yielding cultivars by Cas9, and these mutants showed significant improvement in the yield and cooking quality of rice [114]. From the above findings, we have reached a conclusion that further mutagenesis of amino acid genes could lead to the production of more high-yielding and good quality cultivars in rice. Several rice mutants with high-yielding attributes were generated by editing of *GS3* gene by using Cas9. This study showed that complex traits of rice could be improved using CRISPR/Cas9 in rice [115]. Targeted mutagenesis of *TaSBEIIa* using CRISPR/Cas9 successfully produced high amylose wheat with a meaningfully improved resistant starch content [116]. Alteration of the composition of starch, its structure, and properties via editing of *TaSBEIIa* was studied in spring wheat using CRISPR/Cas9 [116].

The CRISPR/Cas9 technique was used for high-quality oil production, such as oleic acid from *Brassica napus* [117]. CRISPR is used in *Camelina sativa* by changing the function of the *ALCATRAZ INDEHSCENT* and *JAGGED* genes to increase pod shattering resistance [118,119]. In another study [120], the function of *INDEHSCENT* and *ALCATRAZ* genes were affected, which are essential to the dehiscence of fruits in *Brassica* species. Likewise, Zaman, et al. [121] studied the genome editing of *JAGGED* using Cas9 in *Brassica napus* and revealed that this gene was involved in the development of pod shattering resistance. Similarly, the role of *ALCATRAZ* in pod shattering resistance in *Brassica napus* was studied [86]. In vegetables, CRISPR/Cas9 was mainly used in tomatoes because of their economic significance and easy transformation capability. Targeted changes of the engineered gene *SP5G* in tomatoes resulted in early flowering and several brushes, which indicated an early harvest [122]. Liang et al. [54] reported the selective knockout of genes, including *ZmIPK1A*, *ZmIPK*, and *ZmMRP4*, which are involved in the synthesis of phytic acids in maize. The CRISPR/Cas9-based impediment of the *SISG07* gene was studied, and the phenotype of the needle structure in tomatoes was observed [123]. The CRISPR/Cas9 also used to edit the apple protoplast for better taste and quality [124]. CRISPR/Cas9 was used to produce steroidal glycoalkaloids in certain varieties of potatoes to affect *St16Dox*. The study led to the generation of two lines of potatoes free of SGA with a deletion in the *St16Dox* gene [125]. Likewise, the *GBSS* gene that is responsible for the synthesis of starch was mutated using Cas9 in potatoes. An increase in amylopectin levels was observed in mutant lines [126]. The *SnLazy1* locus, known as the *Lazy1* ortholog in tomatoes, was manipulated by CRISPR/Cas9 with effective heritability of the *snlazy-1* allele, and mutants showed a downward pattern of stem development [127]. Many techniques have been developed to improve their yield and quality regarding staple and fruit plants, such as citrus, grapes, and tomatoes, but CRISPR/Cas9 is the most useful and latest method. Earlier studies showed that *Xanthomonas citri*, which advanced the agro infusion method to deliver CRISPR/Cas9, affects the *CsPDS* genetic factors in leaves of sweet oranges [128]. Because of the greater efficiency of genome manipulation, the consumer can now eat edited fruits because this technique does not permit the entry of foreign genes into the genome. Previously, it was reported that the Cas9 technique could be used to edit genes in apple protoplasts, which has been applied in some other crops [124]. A ground cherry of a wild variety of tomatoes yielded higher and larger fruits [129]. Additionally, in apples, a mutation of *PPO* was reflected as transgene-free using Cas9, which is appropriate for human consumption [126]. The production of seedless fruit is always the primary goal of any breeding plan. In tomatoes, the targeting of *SIAGL6* and *SIIAA9* was studied, and parthenocarpy was achieved using Cas9 [127]. The *MaGA20ox2* gene was knocked out in bananas, and the resulting mutation produced dwarf banana genotypes [130]. There are many uses of CRISPR/Cas9 for quality and yield enhancement, which are shown in Table 4.

### 3.2. Development of Disease Resistant Varieties Using CRISPR/Cas9

Plant diseases severely affect crop yields and quality [149];the primary causal agents of diseases are viruses, fungi, nematodes, insects, and bacteria, which cause severe reduction in crop yield. CRISPR/Cas9 is ow widely used for the development of disease-resistant crops [150]. The appearance of deadly outbreaks of these insects and other biotic stresses has become a major issue [151]. Understanding plant and pathogen relationships or interactions are of considerable importance in the defense of plants from these attacks [152]. In the wheat crop, there is an increasing demand for coeliac-safe wheat-based products. Coeliac safe wheat leads to reducing the risk of chronic disorders. These traits cannot be improved by traditional breeding methods. Recently, CRISPR/Cas9 has been used to edit the glutenins genes and produce coeliac safe cultivars. These techniques generate offspring with silenced and deleted gliadins, which may decrease the patients exposure to coeliac disorders (CD) epitopes [153]. Likewise, in another study, Verma, et al. [154] reviewed the role of CRISPR/Cas9 in developing strict-gluten free wheat, which can reduce the risk of coeliac disease (CD). They have concluded that CRISPR/Cas9 has been successfully edited the wheat genome. In the future, this technique would be more useful to develop coeliac-safe wheat rice, the gene *Elf4g* was mutated to increase resistance to thetungro spherical virus in rice [155]. In rice, bacterial leaf blight is a severe problem that causes huge losses in yield. This gene *OsSWEET14* was targeted by Cas9 to induce resistance to the pathogen [156]. This shows that targeted mutagenesis of any susceptible gene could bring significant variation in plant resistance to any pathogen. For instance, the knockout of the *OsERF922* gene was conducted by using Cas9, resulting in increased resistance against blast produced by *Magnaporthe oryzae* [157]. Similarly, the targeted mutation of *SWEET1E* has been performed using Cas9 to produce plants resistant to blight [158]. Therefore, genome editing has been successfully applied to investigate the plant and pathogen relationship, and CRISPR/Cas9 is a potent tool to develop disease-resistant cultivars by mutating the disease-causing gene. For instance, Cas9 was applied to create mutations in the promoter region of the gene *CsLOBI*, which causes citrus canker, and consequently, mutations would result in enhanced resistance to citrus canker. Two mutant lines, *DLoB10* and *DLoB9*, exhibited higher frequencies of mutations. Frameshift mutations and changes in the function of *CsLOBI* resulted in increased resistance to *Xanthomonas citri* [159]. To increase the resistance of citrus against *Xanthomonas*, editing of the effector binding element using Cas9 in the promoter area of the *CsLOBI* gene was performed [160]. The Cas9 technique has been applied to edit the gene *TaMLO* [104] in wheat protoplasts, resulting in the development of enhanced powdery mildew resistance [50]. Similarly, powdery mildew resistance in wheat was developed by targeting *EDRI* homologs using Cas9 [161]. Likewise, *MLO* gene variants were generated in tomatoes, which increased powdery mildew resistance [162]. It was predicted that half of the plant diseases are caused by viruses, which cause substantial losses in crop yield and quality [163]. DNA virus amplicons considerably enhanced the targeting efficiency of the genome. In hexaploid wheat, replicons of geminivirus were used for Cas9 transient expression to counter dwarf virus, and approximately a 12-fold upregulation was noted in gene expression [164]. The geminivirus genome has been targeted by CRISPR/Cas9 and has been used to prevent viral growth [165,166]. Cas9 can be used to edit the viral DNA rather than cure the diseases caused by them [163]. Cas9-mediated alteration of the viral genome can be enhanced by using virus promoters to control sgRNA cassata expression [166]. Recently, a novel ortholog of Cas9 was found in *Francisella novicida*, which was used to manipulate the genome of RNA-based viruses. The *FnCas9* gene helped to halt the replication process of TMV and the cucumber mosaic virus, conferring resistance against them [167]. The targeted mutagenesis of *SIPeLo* and *SIMIO1* was done for trait introgression of tomato using Cas9 to confer resistance against leaf curl virus and powdery mildew [168]. Targeted mutagenesis of *OsPETI* in rice by Casp to improve the resistance to rice sheath blight was studied [169]. A novel diseases resistance paralog using Cas9 has been created in soybean. These novel paralogs confer diseases resistance [170]. Tobacco, an important cash crop, is severely affected by potato virus Y, which is due to the susceptibility of gene *Ntab0942120*. Through the use of CRISPR/Cas9 technique, this gene was knockout and transgene-free homozygous edited plants were generated which showed resistance to *PVY* [171]. In brassica. In an experiment,5genes were knocked out for resistance to Phytophthora in potatoes [138]. Thus, Cas9 is a powerful tool to enhance the genetic makeup of crops to increase their resistance against viruses and other casual agents. A list of crops developed using Cas9 against different diseases is shown in Table 5.

### 3.3. Development of Climate-Smart Crops Using CRISPR/Cas9

Various abiotic stresses have been tackled in major crops, such as rice, wheat, maize, cotton, and potatoes using Cas9. The plant breeding system has been modernized by developing climate-smart or abiotic stress-resistant crops. CRISPR/Cas9 has been used to modify any sequence and to bring any character to crops. Molecular breeders discovered many genes related to abiotic stress tolerance and engineered them into crops [180]. CRISPR/Cas9 generated *slmapk3* protein gene mutants improved defense response to drought stress in tomatoes [181]. Two genes, *TaDREB3* and *TaDREB2*, linked to abiotic stress resistance in wheat protoplasts, have been studied using Cas9. Using the T7 endonuclease assay, mutant gene expression was observed in approximately 70% of the transfected wheat protoplasts. Thus, all mutant plants showed tolerance to drought compared with wild-type plants [182]. A comprehensive review on improvement of drought stress tolerance in plantsusing Cas9 [183]. In rice, two mitogen-activated genes, *OsMKP2* and betaine aldehyde dehydrogenase *OsBADH2*, were manipulated using the Cas9 approach. These genes were delivered into the host genome by protoplast transformation and the particle bombardment technique, which conferred resistance to various stresses [91]. The *OsAnn3* gene of rice was edited against cold stress, and the role of this gene was studied in genome-edited plants [184]. The gene *SAPK2* was altered to examine the stress mechanism in rice. A previous study showed that this gene enhanced drought and salinity tolerance in rice [185]. Drought tolerance has been developed by targeting the *ARGOS8* gene to create new variants, and this is of remarkable significance in maize [186]. Cas9 induced mutagenesis of *Leaf1,2* conferred drought tolerance by affecting the expression pattern of proteinand scavenging of ROS in rice [187]. Zhang et al. [188] enhanced the salinity tolerance in rice via targeted mutagenesis of *OsRR22* gene. Chickpea is an important legume crop that is largely affected by drought stress. Previously only one study has been conducted to bring mutation in genes using CRISPR. Two genes *4CL* and *RVE7* were targeted by Cas9 to increase drought stress tolerance in chickpea. The knockout of these genes using Cas9 is a novel approach that led to the development of drought-tolerant varieties in Chickpea in the future [135]. Recently, de Melo, et al. [189] stated that *AREB-1* activated Arabidopsis by CRISPR presented an enhanced drought tolerance than wild-type plants. CRISPR has also been used to introduce herbicide tolerance in crops. Kuang, et al. [190] studied the base-manipulation facilitated artificial evolution of *OsALS1* in planta to develop new herbicide-tolerant rice germplasm.

Similarly, the Cas-based mutation of two genetic factors, *Drb2a* and *Drb2b*, was studied, and these genes controlled drought and salt tolerance in soybeans [191]. The mitogen-activated protein kinase gene that counteracts drought stress by safeguarding its membrane from oxidative stress and controlling the transcription of genes has been studied to reduce drought stress. The relationship of *SIMPAK3* in drought stress has been studied in tomatoes by producing knockout variants in the *SIMPAK3* gene for the development of drought tolerance via the Cas9 technique [181]. Many genes govern crop yield and stress tolerance. Several studies have been conducted to identify QTLs that govern significant characteristics in crop development systems. Such QTLs have been pyramided into superior lines; however, this is a complex methodfor QTLs closely linked and can produce deleterious effects if non-target regions are transferred. Cas9 can be applied to produce targeted mutagenesis. Using Cas9-based QTL editing approach, two QTLs, *Gn1a* and *GS3*, were investigated in rice varieties [107]. These studies indicate that the CRISPR/Cas9 technique has enormous potential for developing climate-adapted crops. The above studies showed that Cas9 could alter genes to facilitate resistance against many abiotic stresses, e.g., drought, high temperatures, heavy metals, as well as nutrient deficiencies [180]. A list of climate-smart crops is shown in Table 6.

## 4. Novel Breakthroughs

### 4.1. Production of Mutant Libraries

The efficient, practical investigation of all genetic factors of the genome of a sequenced crop is a considerable challenge. The construction of mutant libraries is an effective and reliable method [197,198]. CRISPR/Cas9 is a powerful technique for developing mutant libraries, and its targeting capability can be changed by altering the 18–20 bp target binding order in sgRNA. CRISPR/Cas9 application renders forward genetic screening studies possible. Development of mutant libraries using CRISPR/Cas9 was first reported in human cell culture [198], and this set the basis for CRISPR/Cas9 application to advance plant mutant libraries for high-throughput selection. In an experiment, pooled sgRNA was transformed into tomato plants, and mutants were developed [199]. In rice production, mutant libraries were constructed by two research groups, with loss-of-function mutants generated during the transformation of synthesized sgRNA libraries [197,200]. Certain phenotypic alterations, such as sterility and lethality, were observed when cultivated in the field [197,200]. The general use of CRISPR/Cas9 is valuable for constructing mutant libraries to study the genetic mechanism behindcrop improvement.

### 4.2. Base Editing

CRISPR/Cas 9 is now used to edit a single base, which is accountable for genetic variations in novel traits of crops, as shown by genome-wide association studies [201]. Hence, gene-editing techniques for point mutations are necessary. Without using a DNA repair template, one DNA base can be accurately changed into another DNA base using the CRISPR/Cas9 technique [62]. Cas9 should be fused with an enzyme with conversion capability for base editing. Imidazolinone-resistant rice was produced by base editing, and it is one of the best examples of this technique [202]. In the same manner, *Arabidopsis* was produced [203] by changing *ALS* and a cytidine amino acid-base changer. Similarly, multiple bases were changed in rice [204]. In this approach, the base change tool provides a novel direction for genome editing, widening its potential with specific nucleotide alterations at precise genomes.

### 4.3. Prime Editing

CRISPR has great efficacy to mutate genes but despite its ability to produce accurate base edits outside the four alteration mutations is still the main restraint. Prime editing is another technique of DBS, and it is used to enlarge the area and efficiency of genome editing [205]. This technique hires an engineered reverse transcriptase fused to Cas9 and a prime manipulating guide RNA [205]. This pegRNA varies from sgRNAs as it includes not only the guide order that can identify the objective spots but also a reverse transcriptase template spelling the preferred genetic deviations. Li, et al. [206] newly modified prime editors to bring point alterations, insertions, as well as deletions in rice. By using this method, all 12 kinds of base-to-base replacements, and numerous base replacements, insertions, as well as deletions, were noticed. They stated that the occurrence of prime editing brought by this prime editor was up to 21.8% [206]. Comparable conclusions have been stated by molecular breeders [206,207].

### 4.4. Transgene Free Editing of the Genome

Traditional techniques of genome alterations require the transfer and combination of DNA cassettes encoding altered parts into the host genome. DNA fragments are usually degenerated but produce harmful effects [208]. This technique increases the issues with the regulation of GM plants. Prolonged expression of editing enzymes and systems increases the number of off-target functions because of the greater number of nucleases in these organisms. DNA-free genome editing was first reported in *Arabidopsis* in the same manner as in tomato and rice via delivery of CRISPR/Cas9 RNPs into plant protoplasts [209]. However, methods for effective regeneration of protoplasts do not exist for maximum crops, which leads to the application of particle bombardment and thus facilitates DNA-free genome alteration approaches. In wheat embryos, CRISPR/Cas9 RNAs have been transferred by the particle bombardment technique [144]. 

### 4.5. Multiplex Genetic Engineering 

Using multiple sgRNAs, multiple genomes can be affected simultaneously in any crop. Multiple traits can be incorporated into novel cultivars [210], and multiple individuals from multiple families can be targeted using this technique. This can be attained by combining various sgRNAs into one vector [211]. Clonal reproduction of rice hybrids was previously reported [211]. Hybrid heterozygosity was often fixed via CRISPR/Cas9 genome editing of three genes in three meiotic genes, namely *REC8*, *PAIR1*, and *OSD1*, to generate clonal tetraploid seeds. Hence, multiple GE approaches will provide a more rapid method of creating novel variations in varieties.

## 5. Utilization of CRISPR/Cas9 for Crop Domestication

Domestication of crops and plant breeding led to the development of crops with a high yield that is adjusted to native growing circumstances. Nevertheless, the rising human population faces a number of agricultural challenges, comprising climate alteration, variations in abiotic and biotic stresses, and damage of arable land, alongside a claim for more maintainable and defined agricultural practices. Relatives of modern cultivated and orphans’ crops are considered as an important source of novel variation. However, their low yield and undesirable look prevent their commercial cultivation [212]. Newly, the idea ofde novosubjugation via gene manipulation has been sightseen as a mechanism to bring the wild and orphan crops under domestication rapidly, and therefore advantage from recalled genetic difference and from the features of domesticated plants [213]. This is mainly promising; meanwhile, many classical domesticated genes are perfect candidates for Cas base editing of genes: they are well categorized, have simple genetic architecture, and are monogenetic in nature [214] milarly, ground cherry has alot of unwanted characteristics, like small fruit and a strong stem, which cause fruit dropping. CRISPR base gene-editing technique was used to mutate the gene SP5G, which caused a large number of fruits [129]. These studies prove that Cas-based gene editing can increase the speed of domestication and increase the worth and use of orphan crops.

**Figure 4:**
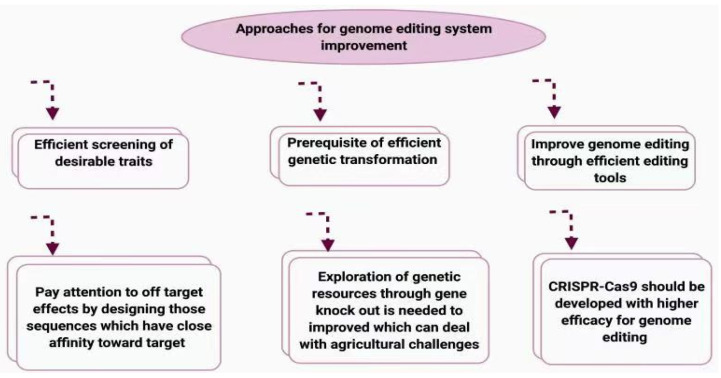
Novel strategies to improve genome edit using Cas9. The editing efficiency of Cas9 could be enhanced by an efficient screening of targeted characters, exploration of genetic material via the knockout of genes, and the use of an ideal gene transformation system.

CRISPR/Cas9 is now being used to domesticate wild plants to serve human needs. Ancient farmers began domesticating all main crops, including rice, wheat, and maize. Nevertheless, our descendants used only a restricted number of originator species during domestication and simply selected plants with better characteristics, such as high yield and ease of breeding, which resulted in a decline in the natural variability of plants. Recalling that genetic diversity is a main concern in the selection process, domestication of wild crops or plants may preserve this diversity. The CRISPR/Cas9 tool has been used to domesticate wild tomatoes, which are tolerant to stress but present with many defaults in fruit production [213]. Six QTLs that were significant for yield were manipulated in one study, and all lines showed enhanced fruit size and fruit number [213]. Many other crops, such as potatoes, bananas, and quinoa, are important locally, have good nutritional value, and are well adapted to local habitats. Despite these features, their low yield and fruit drop prevent their cultivation at larger scales. CRISPR/Cas9 is a powerful tool for manipulating genes and creating desirable crop features. This technique was recently applied to increase flower production and fruit size in ground cherries [129]. With the discovery of genes governing the domestication process, we are confident that we can engineer the genome and increase global food security. To improve the efficiency of genome editing using CRISPR/Cas9, here are the novel techniques by which we can improve it. The first step is we need to do an efficient screening of our desired traits that need to be edited. The knowledge of genetic information about desired traits is a prerequisite in genetic editing, whether it is polygenic or monogenetic. The selection of an efficient tool is important to get good results for editing. Off-target effects should get noticed, and it can be done by designing those sequences which have a close affinity towards each other. The challenges in the agricultural sector are posing a serious threat to crop production. There are many sources of genetic variation which can be utilized and explored using knockout methods to bring desirable changes to meet the need of agricultural crops. The more the efficacy of CRISPR/Cas9, the more the chances of efficient editing will increase, and the greater the desirable results. Novel strategies to improve genome editing using CRISPR/Cas9 are illustrated in Figure 4.

Domestication of crops and plant breeding led to the development of crops with high yields, which are adjusted to native growing circumstances. Nevertheless, the rising human population faces a number of agricultural encounters, comprising climate alteration, variations in abiotic and biotic stresses, and damage of arable land, alongside a claim for more maintainable and defined agricultural practices. Relatives of modern cultivated and orphans’ crops are considered as an important source of novel variation. However, their low yield and undesirable look prevent their commercial cultivation [212]. Newly, the idea ofde novosubjugation via gene manipulation has been sightseen as a mechanism to bring the wild and orphan crops under domestication rapidly, and therefore advantage from recalled genetic difference and from the features of domesticated plants [213]. This is mainly promising; meanwhile, many classical domesticated genes are perfect candidates for Cas base editing of genes: they are well categorized, have simple genetic architecture, and are monogenetic in nature [214].

## 6. Challenges and Limitations of CRISPR/Cas9 Application

CRISPR/Cas9 has several applications in plant breeding, but there are certain limitations. The main challenge is the availability of a small gene pool of important traits; hence, gene availability is important for this tool. Therefore, it is crucial to decode genomic sequence evidence and to investigate valuable genetic resources to improve major crops. It is important to remember that other challenges associated with this technique include the lack of efficient transformation techniques and plant regeneration from cultures that are complex and time-consuming processes. Biosafety issues hinder its application in crop development. Owing to improvements in identification methods, plant mutants with off-target properties can be recognized and detached by separation during successive crosses. In the future, off-target effects using these techniques may be overcome by sgRNAs with high attraction for directed sequences and the choice of Cas9 with high fidelity, comprising good experimental measures. The other chief concern is the issues related to the commercialization of genome-amended crops because these conditions are not encouraging. Recently, the Court of Justice of the European Union stated that genome-amended crops should focus on the same principles as other genetically improved crops [215]. This ruling may delay investment in this technique in the EU. CRISPR has many drawbacks as it does not occur naturally in plants, which means that CRISPR/Cas9 proteins must be moved into plant cells which is a time-consuming process [216] and occasionally needs optimization of codon if Cas9 is from dissimilar backgrounds [217]. The incompetent transfer of CRISPR/dCas9 into the plants is the main obstacle to understanding the prospective of this tool. This is mostly due to the resistance of plant tissue and the incapability of plant tissue to redevelop. Therefore, a new transfer technique like direct transfer of Cas9 constructs into plant apical meristem to circumvent tissue culture is needed [218].

**Figure 5:**
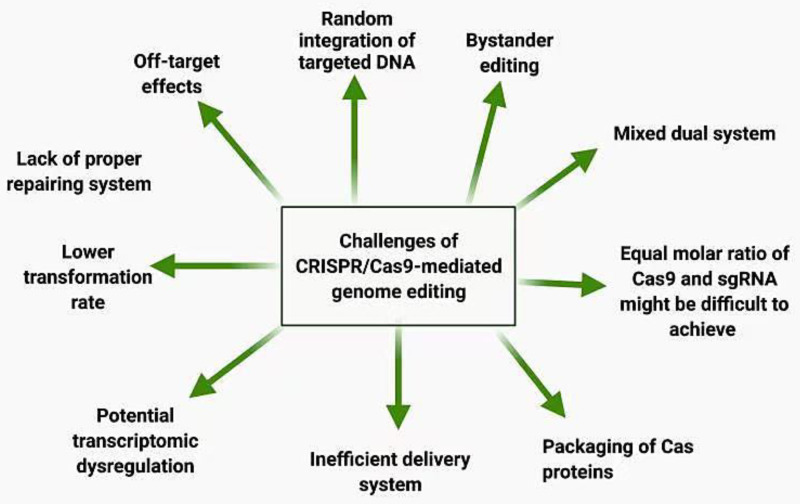
Challenges of CRISPR/Cas9 gene editing. Cas9 has low efficiency of transformation methods, lack of efficiency of the delivery system, and difficultyin achieving an equal molar ratio of Cas9 and sgRNA. These challenges hinder its use for further crops improvement on a larger scale.

With more comprehensive research and more developments in this genome expurgation tool, the CRISPR/Cas9 system may play a significant role in breeding new crop plants for progress toward a sustainable agricultural system that may support a rapidly increasing global population. The efficiency of this technique is restricted because of its large size; thus, it is not suitable for packing into viral vectors for delivery into somatic tissues. For efficient genome editing, the application of CRISPR/Cas9 would yield the best results. CRISPR/Cas9 application can induce numerous accidental off-target changes in the genome [219]. Nevertheless, new CRISPR/Cas9 variants have improved the editing effectiveness of target bases in the sequence desired by the identification of dissimilar PAMs [220]. Problems occur in the commercialization of transgenic crops, thus articulating challenges with CRISPR/Cas9 application in several countries, mainly because of the costs and restrictions imposed by the regulatory system for the field release of genetically altered organisms. Some of the main challenges of CRISPR/Cas9 delivery system are a lack of efficacy or lower efficiency of the delivery system, which limits its use on a larger scale of genome editing. CRISPR/Cas9 based bystander mutations can cause several dysregulations in plants, so this is a huge challenge that needs to be addressed. The presence of off-target effects can minimize our editing efficiency, and this should be reduced using a careful selection of off-target sequences. The off-target effects consist ofunplanned points mutations, deletions, insertions inversions, as well as translocations. Multiple studies using early CRISPR/Cas9 agents revealed that larger than 50% of RNA-guided endonuclease-induced mutations were not generating on-target. Several techniques have been developed to reduce these off-target effects including, biased and unbiased off-target identification, cytosine adenine base manipulators, prime editing as well as truncated called gRNAs [221]. The inefficient Cas9 protein delivery system makes it difficult to reduce the duration of genomic DNA. The unequal molar ratio of sgRNA and CRISPR/Cas9 hinders the use of /CRISPR/Cas9, which is a major limitation as shown in Figure 5. 

## 7. Conclusions and Future Prospects 

The goal of producing safe and low-cost crops to meet the increasing global demands of food using various practices may pose challenges. The use of modern techniques to boost crop varieties will be an essential feature. The use of advanced breeding methods allows scientists to rapidly manipulate genes and insert the gene of interest into the genome compared to classical breeding methods. CRISPR/Cas9 is an essential revolutionary tool for gene editing. Therefore, in the future, the use of this technique to enhance yield, quality, and disease resistance in crops may be a significant field of research. During the last 5 years, it has been applied dynamically in myriad plant systems for conducting practical studies, combating stress-induced responses, and increasing significant agronomic characteristics. However, multiple modifications to this tool should lead to an increase in target effectiveness, and most studies are introductory and warrant development. Nonetheless, CRISPR/Cas9-based genome manipulation will attain a reputation and will be a critical method to attain the generation of “suitably manipulated” plants to help achieve the zero-starvation objective and to realize sustainable food production for the increasing human population. The progress in modern breeding methods has been remarkably accepted as a novelty in our capability to alter genomes and has consequently confronted our consideration of existing regulatory rules. As GE tools are widely used in plants, the protection of GE plants is a problem that warrants discussion worldwide. The regulatory rules for new crop novelties must be multidimensional, precise, and capable of differentiating between GE and genetically modified (GM) crops. Innovative systems biology, next-generation sequencing, and the newest advances in functional genomic methods, cohesive with innovative CRISPR/Cas9 tools, will allow smart crop development with greater yield and enhanced features. The CRISPR/Cas9 tool and speed breeding programs can be used to ensure global food security. CRISPR/Cas9 based genome editing system has the advantage of mixing it with next-generation sequencing. Now researchers can conduct comprehensive mutational screening. Optimization and proper designing of gRNAs are very important at each phase to avoid or reduce the deleterious effects while doing off-target gene editing. Therefore, the use of the CRISPR/CAS9 library has several advantages like high multiplexing, specificity as well as high throughout targeting of a gene. To reduce or nullify negative results, it is important to do a quality check of the CRISPR library at each point during the screening procedure. Analysis of gene function by the above method is critical to recognize the function of genes. Newly exposed CRISPR/Cas9 methods and the development of novel tools are being uninterruptedly described, signifying that CRISPR/Cas9 toolbox for plant engineering will increase further in the future. This set of tools will deliver new methods to achieve defined genome editing without any bits of transgenes residual in genome-edited plants.

## Figures and Tables

**Table 1:** Use of MNs, ZFNs, and TALENs for crops improvement.

Crop	Tool	Gene	Trait	Reference
Rice	TALEN	*OsBADH2*	Fragment rice	[48]
Rye	Cas9	*TrpE*	Fungal resistance	[49]
Wheat	TALEN	*TaMLO*	Resistance to powdery resistance	[50]
Maize	MNs	*LGI*	Targeted mutagenesis	[51]
Cotton	EMNs	*EPSPS*	Tolerance to herbicide	[38]
Tobacco	TALENs	*Sur A*	Directed mutation	[52]
Barley	TALENs	*Transgene*	Resistance to powdery resistance	[53]
Maize	TALENs	*ZMIPK*	Phytic acid biosynthesis Liang	[54]
Potato	TALENs	*StGBSS*	Quality of tuber starch	[55]

**Table 3:** RNA tools developed for CRISPR/Cas9.

Tool	Function	Year	Reference
tracr RNA	Act as reference point	2021	[76]
CRISPR design	RNA construction for targeted regions and assessment of off target effects	2013	[77]
Cas12j	Genome manipulation	2021	[78]
sgRNAcas9	Speedy construction of sgRNA and fewer effects	2014	[79]
CCTop	Estimate target sgRNA sequence on the basis of off-target influences	2015	[80]
phytoCRISP.Ex	Cas9 target prediction	2016	[81]
Cas12	Target recognition	2021	[82]
sgRNA Scorer 2.0	sgRNA construction for many PAM locates	2017	[83]
CRISPR-Local	sgRNA construction for non-reference types	2018	[84]
CRISPRInc	Construct sgRNA for lncRNAs	2019	[85]

**Table 4:** Uses of CRISPR/Cas9 for quality and yield enhancement in crops.

Gene	Crops	Trait	Technique	Reference
*CLE*	Maize	Grain yield	CRISPR/Cas9	[131]
*SLIAA9*	Tomato	Seedless fruit	CRIISPR/Cas9	[132]
*OsTB1*	Rice	High grain yield	CRIISPR/Cas9	[114]
*GmFT2a*	Soybean	Delay in flowering time	CRIISPR/Cas9	[133]
*BnITPK*	Rapeseed	Low phytic acid	CRIISPR/Cas9	[134]
*Bolc.GA4.a*	Cabbage	Dwarf plant	CRIISPR/Cas9	[106]
*OsAAP10*	Rice	Good cooking quality	CRIISPR/Cas9	[114]
*RVE7*	Chickpea	Grain quality	CRIISPR/Cas9 B	[135]
*BnaMAXI*	Rapeseed	Yield	CRISPR/Cas9	[136]
*Wholek1gene*	*Sorghum*	Increase lysine content	CRIISPR/Cas9	[137]
*TaSBEIIa*	Wheat	High amylose content	CRIISPR/Cas9	[116]
*GBSS*	Potato	Enhance amylose content	CRIISPR/Cas9	[138]
*P450*	Rice	High grain yield	CRIISPR/Cas9	[113]
*IPA*	Rice	Enhanced yield	CRIISPR/Cas9	[139]
*CIPDS*	Watermelon	Albino phenotype	CRIISPR/Cas9	[140]
*GS3*	Rice	Grain yield	CRIISPR/Cas9	[115]
*ANTI*	Rice	Fruit color	CRIISPR/Cas9	[141]
*OsSPL16*	Rice	Grain yield	CRIISPR/Cas9	[108]
*lncRNA1459*	Tomato	Prolong shelf life	CRIISPR/Cas9	[142]
*PRL*	Maize	Reduction in zein content	CRIISPR/Cas9	[143]
*GASR7*	Wheat	Grain weight	CRIISPR/Cas9	[144]
*PDS*	Banana	Albino phenotype	CRIISPR/Cas9	[145]
*CsFAD2*	Camelina	Oleic acid	CRIISPR/Cas9	[146]
*FaTM6*	*Strawberry*	Flower development	CRIISPR/Cas9	[147]
*GmFATB1*	Soybean	Low saturated fatty acid	CRIISPR/Cas9	[148]

**Table 5:** A list of diseases resistant crops varieties made by using CRISPR/Cas9.

Gene	Chromosomal Position	Locus	Pathogen	Crop	Function	Trait	Repair Tool	Editing Results	Reference
*SIPeLo* and *SIMIO1*				Tomato	Enhance resistance to leaf curl virus	Enhance resistance to leaf curl virus			[168]
*OsSWEET14*				Rice	Bacterial leaf blight	Bacterial leaf blight resistance			[156]
*CsLOB1*	N/A	N/A	Xanthomonas citri	Citrus	Susceptibility to citrus canker	Resistance to citrus canker	NHEJ	Knockout	[159]
*Gh14-3-3d*	https://www.ncbi.nlm.nih.gov/nuccore/164652939 (accessed on 15 October 2020)	N/A	Verticillium dahliae	Cotton	Negative controller of resistance to disease	Verticillium wilt resistance	NHEJ	Knock-in	[172]
*Ntab0942120*				Tobacco	Resistance to potato virus Y	Resistance to potato virus Y			[171]
*eLF4G*	chr07:22114961..22123061 (+ strand)	Os07g0555200	Tungro spherical virus	Rice	Starting factor for initiation	Resistance to tungro spherical virus	NHEJ	Knock out	[155]
*Rpsl*				Soybean	Diseases resistance	Diseases resistance			[170]
*Rp and Cp sequences*	N/A	N/A	Yellow leaf curl virus	Tomato	Negative controller of resistance	Enhanced resistance again leaf curl virus	NHEJ	Knock out	[173]
*OsSWEET11*	chr08:26725952..26728794	Os08g0535200	Bacterial blight	Rice	Resistance to bacterial blight	Resistance to bacterial blight	NHEJ	Knock out	[174]
*OsSWEET14*	chr11:18171707..18174478	Os11g0508600	Bacterial blight	Rice	Resistance to bacterial blight	Resistance to bacterial blight	NHEJ	Knock out	[175]
*SiMLOl*	N/A	N/A		Tomato	Powdery mildew resistance gene	Resistance to powdery mildew	NHEJ	Knock out	[162]
*Jaz2*	Chr12: 2502581..2504643	N/A	Pseudomonas syringae	Tomato	Bacterial speck resistance	Bacterial speck resistance	NHEJ	Knock out	[176]
*WRKY70*	LK032201:212360-212875	BnaA09g35840D		Brassica		Resistance to pathogens	NHEJ	Knock out	[177]
*MYB28*				Brassica	Glucoraphanin accumulation		NHEJ	Knock out	[178]
*S-genes*	N/A	N/A		Potato	Resistance to Phytophthora	Resistance to Phytophthora	NHEJ	Knock out	[138]
*TaMLO-A1*	Chromosome 4A: 519,570,414-519,575,284	TraesCS4D02G318600		Wheat	*Resistance to mildew*	*Resistance to mildew*	NHEJ	Knock out	[50]
*RGA2, Ced9*	3:6700445-6700466	GSMUA_Achr3G09290_001	*Fusarium oxysporum*	Banana	*Resistance to fusarium wilt*	*Resistance to fusarium wilt*	*NHEJ*	Knock out	[162]
*Mlo-7*	Chr13: (5335059..5339258	VIT_00016304001		Grape	*Resistance to mildew*	*Resistance to mildew*	*NHEJ*	Knock out	[124]
*PpalEPIC8*	N/A	N/A	*Phytophthora palmivora*	Papaya	*Resistance to Phytophthora*	*Resistance to Phytophthora*	*NHEJ*	Knock out	[179]

**Table 6:** Development of climate smart crops using CRISPR/Cas9.

Gene	Chromosomal Position	Locus	Crop	Traits	Repair Pathway	Editing Results	References
*OsMYB30*			Rice	Cold tolerance			[115]
*slmapk3*			Tomato	Drought tolerance			[181]
*OsALS1*			Rice	Herbicide tolerance			[190]
*4CL, RVE7*			Chickpea	Drought tolerance			[135]
*SAPK2*	chr07:25717837..25722009 (+strand)	Os07g0622000	Rice	Tolerance to salinity and drought	NHEJ	Knockout	[185]
*TaDREB3*	7D:27470774-27471448	TraesCS7D02G052600	Wheat	Tolerance to drought	NHEJ	Knockout	[182]
*NPRI*	3:3740543-3741413	Solyc03g026270.2	Tomato	Tolerance to cold and drought stress	NHEJ	Knockout	[142,192]
*ZmHKTI*	N/A	N/A	Maize	Tolerance to salinity stress	NHEJ	Knockout	[193]
*Drb2a*	12:5797459-5798459, 11:11249371-11250406	GLYMA_12G075700 GLYMA_11G145900	Soyabean	Tolerance to drought and salinity stress	NHEJ	Knockout	[191]
*OsRR22, OsPDS*	chr10:17076098..17081344 (- strand) chr03:4410090..4414779 (+ strand)	Os10g0463400 Os03g0184000 (OsPDS)	Rice	Tolerance to salinity stress	NHEJ	Knockout	[188,194]
*OsAOX1a,*	chr04:30287197..30289860	Os04g0600200	Rice	Drought resistance	NHEJ	Knockout	[104]
*OsBADH2*	chr08:20379823..20385975	Os08g0424500	Rice	Abiotic stress resistance	HDR	Knockout	[91]
*ALS1*	chr3:8175606-8177917	PGSC0003DMG400034102	Potato	Resistance to herbicide	HDR	Knockout	[195]
*MIR169a*	Chr03:4358994-4359219	AT3G13405	Arabidopsis	Drought resistance	HDR	Knockout	[196]

## Data Availability

Not applicable.
